# In Sickness and in Health: Dyadic Perspectives on Health and Well-Being Among Older Couples

**DOI:** 10.1093/geroni/igaa057.2232

**Published:** 2020-12-16

**Authors:** Courtney Polenick

## Abstract

This session will incorporate a variety of dyadic methods to explore the multifaceted ways that older spouses shape their own and their partners’ health and well-being. First, Dr. Karen Lyons will consider the roles of communication, collaborative decision-making, and social support in shaping the mental health of couples managing chronic pain. Her comparative dyadic analysis highlights the value of collaborative illness management in optimizing couples’ mental health. Dr. Courtney Polenick will then describe how chronic condition discordance (i.e., the extent to which two or more conditions have non-overlapping self-management requirements) within individuals and between spouses is linked to perceived control among couples over an8-year period. This study reveals that more complex patterns of chronic conditions within couples have particularly detrimental implications for women’s perceptions of control over their own health and other life domains. Next, Dr. Kira Birditt will examine the long-term effects of spouses’ similar drinking patterns (i.e., concordance). Although drinking concordance may enhance marital satisfaction, she will explain how it can have enduring negative consequences for cardiovascular health among middle-aged men. Dr. Joan Monin will then explain the short-term benefits of laughter for blood pressure among couples during lab-based spousal support interactions. Finally, Dr. Amy Rauer will discuss how spouses react to one another’s health-related support attempts using in-depth qualitative interviews conducted with both members of the couple. Taken together, these studies underscore the importance of evaluating dynamic short-term and long-term health-related influences among couples in middle and later life. Dyadic Research on Health and Illness Across the Adult Lifespan Interest Group Sponsored Symposium.

